# Bioremediation of soils saturated with spilled crude oil

**DOI:** 10.1038/s41598-019-57224-x

**Published:** 2020-01-24

**Authors:** Nedaa Ali, Narjes Dashti, Majida Khanafer, Husain Al-Awadhi, Samir Radwan

**Affiliations:** 1 Microbiology program, Department of Biological Sciences, Faculty of Science, Kuwait University, P.O.Box 5969, Safat, 13060 Kuwait; 2 Von Einem Str. 25, 48159 Münster, Germany

**Keywords:** Bacteriology, Soil microbiology

## Abstract

A desert soil sample was saturated with crude oil (17.3%, w/w) and aliquots were diluted to different extents with either pristine desert or garden soils. Heaps of all samples were exposed to outdoor conditions through six months, and were repeatedly irrigated with water and mixed thoroughly. Quantitative determination of the residual oil in the samples revealed that oil-bioremediation in the undiluted heaps was nearly as equally effective as in the diluted ones. One month after starting the experiment. 53 to 63% of oil was removed. During the subsequent five months, 14 to 24% of the oil continued to be consumed. The dynamics of the hydrocarbonoclastic bacterial communities in the heaps was monitored. The highest numbers of those organisms coordinated chronologically with the maximum oil-removal. Out of the identified bacterial species, those affiliated with the genera *Nocardioides* (especially *N*. *deserti*), *Dietzia* (especially *D*. *papillomatosis*), *Microbacterium*, *Micrococcus*, *Arthrobacter*, *Pseudomonas*, *Cellulomonas*, *Gordonia* and others were main contributors to the oil-consumption. Some species, e.g. *D*. *papillomatosis* were minor community constituents at time zero but they prevailed at later phases. Most isolates tolerated up to 20% oil, and *D. papillomatosis* showed the maximum tolerance compared with all the other studied isolates. It was concluded that even in oil-saturated soil, self-cleaning proceeds at a normal rate. When pristine soil receives spilled oil, indigenous microorganisms suitable for dealing with the prevailing oil-concentrations become enriched and involved in oil-biodegradation.

## Introduction

Pollution with crude oil and its products has become globally a major environmental concern. At least 0.08 to 0.4% of the internationally produced oil has been estimated to be spilled in the marine ecosystem as pollutants^1^. Considering that also the terrestrial and atmospheric ecosystems receive yearly increasing amounts of spilled oil, it could be imagined how serious this environmental problem is.

In view of the fact that the Arabian/Persian Gulf region produces more than 50% of the marine transported oil in the world, the Gulf countries actually receive a big share of oil-pollution. The greatest man-made oil spill was associated with the Second Gulf-War (1990–1991), when the Iraqi forces damaged and set in fire about 700 Kuwaiti oil wells, and released from the Mina Al-Ahmadi Terminal into the Gulf water body crude oil through 3 successive days^2^. Being rather resistant to biodegradation, oil-pollutants persist in the environment^3^. Under normal conditions, oil in soil persists much longer than most conventional carbon sources, e.g. carbohydrate and proteins, which take only weeks to be degraded. Under extreme conditions on the other hand, e.g. drought, oil persists much longer. The oil lakes that resulted from the 700 wells damaged by the Iraqis during their withdrawal in February 1991 still contain the oil undegraded in the form of heavy sediments. Further, aromatic oil-constituents are toxic compounds^4^.

Although physical and chemical approaches for the removal of spilled oil are sometimes in use^5,6^, they are mostly neither cost-effective nor environmentally friendly^7^. For example, incineration leads to air pollution and land-filling to contamination of ground waters^5^. For incineration, the spilled oil is simply burned, with the consequences of raising the atmospheric carbon dioxide-, nitrogen oxide- and sulfur oxide-levels. The current problem of global warming is known to be due to the accumulation of CO_2_ in the atmosphere. It may be argued that also bioremediation leads to the release of CO_2_ by bacteria. In fact, only one part of the oil’s carbon is released during energy (ATP) production by bacteria, but the other part is conserved in the soil as bacterial cell material. Nitrogen and sulfur oxides are responsible for the acidic rain. Land-filling was reported to produce hazardous leachates in the form of gases and liquids which potentially toxify the ground water^8,9^. Surely, those approaches lead to removal of substantial proportions of the spilled oil. However, the unpredictable hazards associated with their use are certainly serious limitations for their implementation. More about such hazards are available in recent reviews^10,11^.

On the other hand, oil-bioremediation^12–14^ is globally recognized as a cost effective and environmentally safe approach. Bioremediation comprises two distinct operations, bioaugmentation (seeding, inoculation) and biostimulation. Bioaugmentation implies the inoculation of exogenous microorganisms into the polluted site^15,16^. In other words, this approach results in the addition of more gene pools into the contaminated sites^17^. Biostimulation, on the other hand, relies on the already existing (native) microorganisms which may be enhanced in their activities via specific managements. Most frequent among those managements is the fertilization with N- and P- compounds^18–20^. Depending in oil-removal on the already existing microorganisms without any specific management is termed “self-cleaning”.

In most of the bioremediation studies, only relatively low concentrations of crude oil (1–6%) have been used^21–25^. However, the desert areas of Kuwait polluted with oil since the 1991 War contain much higher proportions of oil; up to 20% or more^2^. Reportedly, the crude oil that spilled from the damaged wells filled about 50 “oil-lakes” of varying dimensions. Through interference with the soil aeration and water retention, and due to the toxicity of oil constituents^26–28^, life of lower and higher organisms becomes difficult or impossible at such high oil concentrations.

The objective of this work is to study whether effective microbial-biodegradation of oil would proceed in oil-saturated soils, and whether diluting the oil concentration by mixing such oil-saturated soils with oil-free soil would enhance the oil-bioremediation process. There are no earlier studies published in the available literature on this subject. To fulfill this objective, a desert soil sample was artificially polluted with 17.3% oil, the amount of oil needed to saturate this soil. Further, samples of this polluted soil were diluted to various degrees with clean soil samples. The fully polluted and diluted samples were incubated under open conditions for six months after which they were analyzed for the proportions of oil consumed as well as for the hydrocarbonoclastic bacterial communities. Comparison of the results was expected to answer the raised questions.

## Results

### Oil-removal during bioremediation

The oil-removal values in the oil-saturated and diluted desert soil samples are presented in Table 1. Between 16 and 18% of the 173 g in each heap has been lost during the adaptation month of March. More than one half of the oil, namely 53 to 63% was removed by the end of the first month, April, of bioremediation. Oil-removal continued, albeit at slower rates during the subsequent five months. At the end of September, only 37 to 47 out of the 173 g oil remained undegraded in each heap. This corresponds to final oil-removal values of 73–79%. The statistical analysis revealed that all the above oil-removal values were significant (ANCOVA, n = 3, *p* < 0.05). On the other hand, there were no significant differences in the oil-removal values between the oil-saturated and the diluted oily soil samples (ANCOVA, n = 3, *p* > 0.05).

**Table 1:** Oil-consumption in oil-saturated desert soil (OSS) and OSS diluted to different degrees with pristine desert- and garden- soils.

End of the month (min-max °C)		Oil-saturated soil (OSS)		OSS diluted with pristine desert soil						OSS diluted with pristine garden soil					
				I		II		III		I		II		III	
		A	B	A	B	A	B	A	B	A	B	A	B	A	B
0	March (22–34)	144 ± 12.3	17	146 ± 1.5	16	ND	ND	142 ± 4.7	18	145 ± 3.4	16	ND	ND	ND	ND
1	April (26–44)	71 ± 3.1	59	64 ± 3.4	63	74 ± 3.6	57	75 ± 0.6	57	75 ± 1.0	57	81 ± 10.0	53	82 ± 8.1	53
2	May (34–46)	62 ± 1.4	64	53 ± 3.8	69	56 ± 6.1	68	60 ± 0.8	65	65 ± 2.4	62	62 ± 5.8	64	61 ± 5.3	65
3	June (39–50)	48 ± 7.1	72	48 ± 0.8	72	54 ± 0.2	69	51 ± 0.5	71	40 ± 0.7	77	54 ± 6.2	69	54 ± 10.5	69
4	July (44–51)	47 ± 1.7	73	41 ± 1.8	76	50 ± 3.4	70	46 ± 2.9	73	43 ± 0.05	75	48 ± 6.0	72	40 ± 8.0	77
5	August (42–51)	44 ± 0.51	74	40 ± 0.8	77	39 ± 0.4	77	40 ± 0.5	78	40 ± 1.0	77	44 ± 4.4	75	40 ± 6.5	77
6	September (39–47)	42 ± 1.9	76	39 ± 3.2	77	37 ± 2.2	79	47 ± 5.6	73	39 ± 1.5	77	42 ± 3.1	76	38 ± 5.1	77

I, II and III; 1 kg OSS mixed with 0.25, 0.50 and 0.75 kg pristine soil, respectively; A, gram residual oil per heap (initial concentration, 173 g in all heaps) ± standard deviation values; B, % oil consumed based on the initial concentration; ND, not determined. Samples were taken at the ends of the adaptation month (0 March) and of the 6 subsequent months.

### The hydrocarbonoclastic bacterial communities

The individual bacterial strains reported in this paper were counted and isolated on a mineral medium with oil-vapor as the sole source of carbon and energy (selective medium, see the section: Materials and Methods), which implies that all the strains are hydrocarbonoclastic. The data in Table 2 show that the CFU numbers of oil-utilizing bacteria in the oil-saturated and diluted soil samples at the end of the adaptation month of March were in the magnitude of one to three million CFU per gram soil. One month after the start of the bioremediation experiment, i.e. at the end of April, the numbers increased significantly (ANOVA, n = 3, *p* < 0.05) reaching the magnitudes of tens to hundreds of millions of CFU per gram soil. The highest count of 4.9 × 10^8^ CFU g^−1^ was recorded in the oil-saturated soil sample. The numbers then decreased significantly with time reaching the magnitudes of millions to ten-millions of CFU g^−1^ (ANOVA, n = 3, *p* < 0.05). In the hot months of July, August and September, relatively higher counts in the magnitude of hundred million CFU g^−1^ were obtained again, especially in the pristine desert- and garden-soil diluted desert heaps. Using ANCOVA, there were no significant difference in the CFU numbers among the analyzed soil samples (ANCOVA, n = 3, *p* > 0.05) once their means had been adjusted for time.

**Table 2:** Numbers of CFU of hydrocarbonoclastic bacteria in oil-saturated desert soil (OSS) and OSS diluted with pristine desert and garden soils.

End of month	Outdoor temperature (min-max °C)	Numbers of CFU g^−1^ (×10^6^) ± standard deviation values							
		Oil-saturated soil (OSS)		OSS diluted with pristine desert soil			OSS diluted with pristine garden soil		
				I	II	III	I	II	III
0	March	22–34	2.7 ± 0.1	1.7 ± 0.1	1.9 ± 0.1	1.8 ± 0.05	1.3 ± 0.1	1.3 ± 0.05	1.1 ± 0.0
1	April	26–44	487.5 ± 16.2	164.0 ± 12.2	38.3 ± 2.1	36.3 ± 1.1	31.0 ± 2.1	70.0 ± 2.0	356.0 ± 20.0
2	May	34–46	10.5 ± 1.0	15.7 ± 0.9	14.4 ± 1.0	76.5 ± 4.0	15.8 ± 1.2	22.3 ± 1.1	64.5 ± 11.1
3	June	39–50	4.1 ± 0.8	1.7 ± 0.05	3.7 ± 1.0	11.4 ± 1.0	4.2 ± 0.5	38.0 ± 2.4	17.5 ± 2.1
4	July	44–51	0.9 ± 0.0	2.5 ± 0.08	25.4 ± 1.2	153.0 ± 12.1	84.5 ± 2.3	69.5 ± 5.1	114.0 ± 6.2
5	August	42–51	0.8 ± 0.05	1.0 ± 0.0	56.5 ± 2.0	125.0 ± 10.0	18.5 ± 1.5	197.0 ± 10.2	386.5 ± 15.3
6	September	39–47	1.7 ± 0.1	1.2 ± 0.06	55.7 ± 2.0	128.1 ± 10.0	14.2 ± 1.0	110.0 ± 11.0	58.5 ± 5.3

I, II and III; 1 kg OSS mixed with 0.25, 0.50 and 0.75 kg pristine soil, respectively.

### Dynamics of the hydrocarbonoclastic bacterial communities in the soil diluted with pristine desert soil

**Figure 1:**
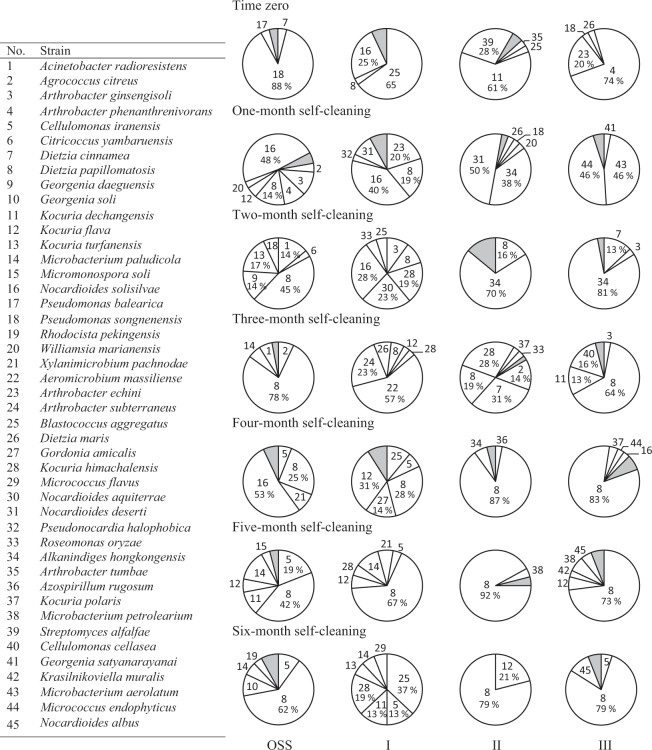
Dynamics of hydrocarbonoclastic microbial communities in the oil-saturated soil (OSS) and OSS diluted (with pristine desert soil: I, 1 kg OSS + 0.25 kg; II, 1 kg OSS + 0.5 kg; III, 1 kg OSS + 0.75 kg) during bioremediation. Shaded areas contain all the bacterial strains with ≤2% occurrence. For minor organisms in the shaded areas, refer to Supplementary Table S2.

Figure 1 shows the composition of the hydrocarbonoclastic bacterial communities in the various heaps through the six-month-bioremediation. The oil-saturated soil (OSS) that had been stabilized for one month (end of March, zero-reading) contained *Pseudomonas songnenensis* as the predominant species. One month after the experimental set up (end of April), the predominance was taken over by *Nocardioides solisilvae* (48%), *Dietzia papillomatosis* (14%) and two *Arthrobacter* spp (21%). *D*. *papillomatosis* persisted till the end of the experiment as the most dominant or one of the predominant taxa in the oil-saturated heap. Another species which shared the predominance at later phases of bioremediation was *Nocardioides solisilvae*.

The lowest grade of dilution of this soil with pristine desert soil (heap I) resulted in the following pattern of predominance. End of March and April, *Blastococcus aggregatus* (65%) and *Nocardioides solisilvae* (40%) prevailed and *Dietzia papillomatosis* (19%) started to appear. End of May, *Nocardioides solisilvae* (28%), *N*. *aquiterrae* (23%), and *Kocuria himachalensis* (19%) shared the predominance, while end of June, *Aeromicrobium erythreum* (57%) and *Arthrobacter subterraneus* (23%) became predominant. End of July, *Kocuria flava* (31%) and *Dietzia papillomatosis* (28%) shared the predominance and end of August, *Dietzia papillomatosis* (67%), was absolutely dominant. End of September, *Blastococcus aggregatus* (37%), *Kocuria himachalensis* (19%), *Kocuria dechangensis* (13%) and *Cellulomonas iranensis* (13%) prevailed.

The moderate-grade dilution sample (heap II) showed the following predominance patterns. End of March, *Kocuria dechangensis* (61%) and *Streptomyces alfalfae* (28%) were predominant and end of April, *Nocardioides deserti* (50%) and *Alkanindiges hongkong*ensis (38%) took over the predominance. End of May, *Alkanindiges hongkong*ensis (70%) was absolutely predominant and *Dietzia papillomatosis* (16%) started to appear. End of June, *Dietzia cinnamea* (31%), *Kocuria himachalensis* (28%) and *Dietzia papillomatosis* (19%) shared the predominance. End of July till end of September, *Dietzia papillomatosis* took over the absolute predominance.

The highest grade of sample (heap III) showed the following predominance patterns. End of March, two *Arthrobacter* species (94%) shared the absolute predominance. End of April, *Microbacterium aerolatum* and *Micrococcus endophyticus* (46%, each) prevailed. End of May, *Alkanindiges hongkong*ensis (81%) and *Dietzia cinnamea* (13%) shared the predominance. End of June till end of September, *Dietzia papillomatosis* took over the absolute dominance.

### Dynamics of the hydrocarbonoclastic bacterial communities in the soil diluted with pristine garden soil

**Figure 2:**
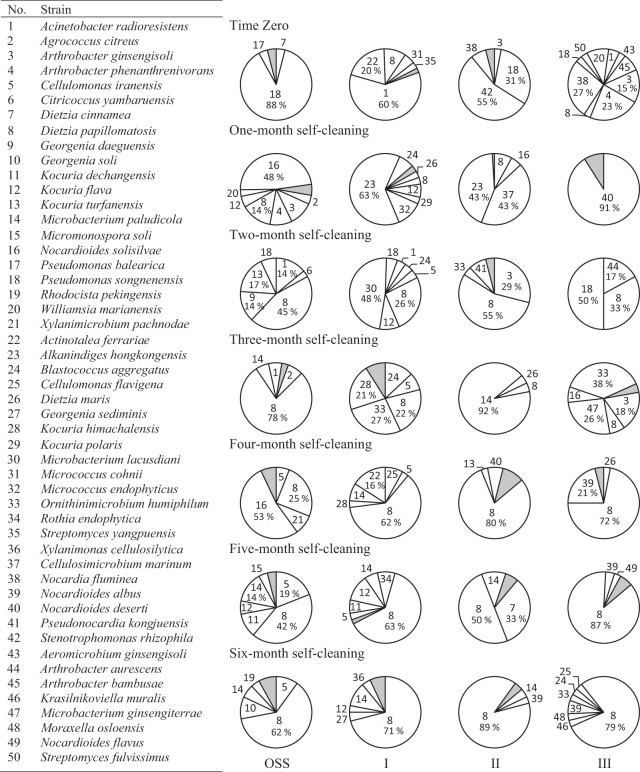
Dynamics of hydrocarbonoclastic microbial communities in the oil-saturated soil (OSS) and OSS diluted (with pristine garden soil: I, 1 kg OSS + 0.25 kg; II, 1 kg OSS + 0.5 kg; III, 1 kg OSS + 0.75 kg) during bioremediation. Shaded areas contain all the bacterial strains with ≤2% occurrence. For minor organisms in the shaded areas, refer to Supplementary Table S2.

Figure 2 presents the dynamics of the hydrocarbonoclastic communities in the three heaps. The predominance patterns in the oil-saturated soil have been described above, and are repeated in Fig. 2 to facilitate the comparison. The lowest grade of dilution sample (heap I) showed the following patterns of predominance. End of March, *Acinetobacter radioresistens* (60%) was predominant, but end of April, *Alkanindiges hongkongensis* (63%) took over the predominance. End of May, *Microbacterium lacusdiani* (48%) and *Dietzia papillomatosis* (26%) predominated. End of June, *Ornithinimicrobium humiphilum* (26%), *Dietzia papillomatosis* (22%) and *Kocuria himachalensis* (21%) shared the predominance. End of July till end of September, *Dietzia papillomatosis* (71%) took over the absolute predominance.

The moderate grade of dilution sample (heap II) showed the following predominance patterns. End of March, *Stenotrophomonas rhizophila* (55%) and *Pseudomonas songnenensis* (31%) were predominant. End of April, *Cellulosimicrobium marinum* and *Alkanindiges hongkongensis* shared the predominance (43%, each) over *Dietzia papillomatosis* (8%). End of May, *Dietzia papillomatosis* (55%) prevailed. End of June, *Microbacterium paludicola* (92%) took over the absolute predominance. End of July till end of September, *Dietzia* species, particularly *D. papillomatosis* took over the absolute predominance.

The highest grade of dilution sample (heap III) showed the following predominance patterns. End of March, *Nocardia fluminea* (27%) prevailed together with three *Arthrobacter* species (46%). End of April, *Nocardioides deserti* took over the absolute dominance (91%). End of May, *Pseudomonas songnenensis* (50%) and *Dietzia papillomatosis* (33%) shared the predominance. End of June, *Ornithinimicrobium humiphilum* (38%) and *Microbacterium ginsengiterrae* (26%) prevailed. End of July till end of September, *Dietzia papillomatosis* took over the absolute dominance.

In addition to those predominant species in Figs. 1 and 2, many other hydrocarbonoclastic bacterial species occurred in the analyzed samples as less dominant constituents (Supplementary Table S1). Table S2 in the Supplementary information includes data about the sequencing of the individual isolates and their accession numbers in the GenBank database.

### Oil-tolerance of pure isolates

**Figure 3:**
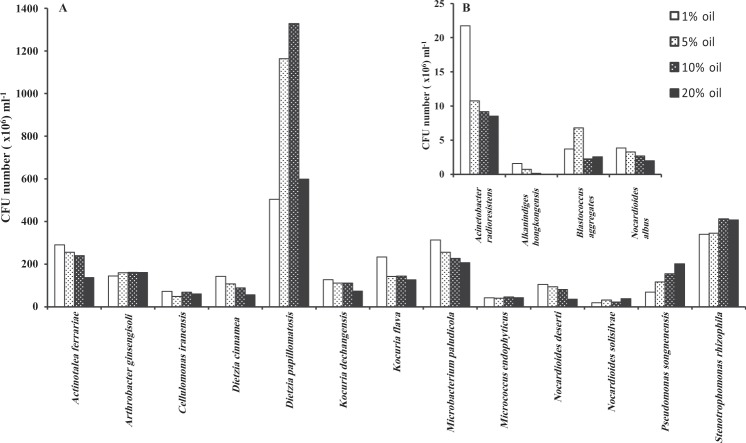
Oil-tolerance of 17 hydrocarbonoclastic isolates. (**A**) strains with highest tolerance; (**B**) strains with less tolerance.

Figure 3 shows that the seventeen tested representative isolates could be divided into two groups: group A which included the thirteen isolates with the highest oil-tolerance, and group B the four isolates with weaker tolerance. Nevertheless, the mere capability of growth in the presence of up to 20% oil as a sole carbon source implies that all the isolates survived and even propagated in the presence of 20% oil. The isolate with the highest tolerance potential was *Dietzia papillomatosis*. Group A comprised members whose growth was enhanced with increasing oil-concentration, whereas the growth of members of group B was relatively weaker at the highest oil-concentrations.

Broad beans seeds failed to germinate in the untreated oil-saturated desert soil but showed 96% germination in the 6-month bioremediated soil and the seedlings developed to maturity (Supplementary Figure S1).

## Discussion

The finding that self-cleaning of an oil-saturated soil (17.3%, w/w) proceeds as effectively as that of the same soil charged with much less concentrations is novel and interesting for basic science and from the practical viewpoint. We did not find any similar bioremediation studies in the available literature on oil-saturated soils. As mentioned in the introduction, the oil-concentrations used by earlier researchers ranged between 1 and 6% only^21–25^. This new result raises important questions that need answers by basic scientists. How can bacteria live, propagate and metabolize at such an oil concentration which interferes with aeration and availability of water and nutrients^29^? How can they resist the toxic effects of oil-constituents particularly the aromatics^28,30^?. This paper does not present data that may contribute to answering those difficult questions. However, we may recall that the growth of hydrocarbonoclastic microorganisms in aircraft fuel reservoirs is a well known problem that should be avoided for security reasons. Moreover, soil microorganisms naturally live and survive in microenvironments. Therefore, aerophobic bacteria for example are routinely isolated from well aerated soils, obligate acidophiles from neutral soils and thermophiles from soils in temperate and arctic regions. Within this context, Benyahia *et al*.^31^, working with a biopile system, reported that oil in a soil sample treated with bacterial products occurred as thin films on tiny soil particles and that bacterial products created particle aggregates with channels for air and water flow within the bulk soil. From the practical view point, it is interesting to know that bioremediation proceeds also in very heavily contaminated sites. Obviously, the high oil content leads automatically to enriching the soil selectively with microbial species with extremely high oil concentration tolerance. The results of this study provide evidence for this, and demonstrate that *Dietzia papillomatosis* is the most prominent example to be named, here.

To simulate field-bioremediation, we had to leave the soil heaps exposed to the open atmosphere. No doubt, a proportion of oil must have been lost by volatilization. However, the fact that only 16 to 18% of the oil was lost during March, the month of microbial adaptation and stabilization (Table 1), indicates that the loss through volatilization although significant was rather limited.

The data in Tables 1 and 2 provide an experimental evidence for the effective involvement of the bacterial flora in the consumption of oil. Chronologically with maximum oil-removal, there were in all the studied samples peaks for the hydrocarbonoclastic bacterial counts. There were also increases in bacterial counts during the hot months of July and August, probably due to the enhanced activity of thermophilic/thermotolerant bacteria. All the strains were isolated on a selective mineral medium with crude oil as a sole source of carbon and energy, i.e. they all were hydrocarbonoclastic. In contrast to conventional species, the hydrocarbonoclastic strains posses mono- and dioxygenases, which catalyze splitting the oxygen molecule into atoms and introducing the latter into aliphatic and aromatic hydrocarbons, respectively. This initial attack on hydrocarbons leads, e.g. to the production of alkanols from alkanes, which are subsequently oxidized via alkanals to the corresponding fatty acids. The latter are degraded by β-oxidation to Acetyl-CoA units. Through the Kerbs cycle, this intermediate becomes mineralized to CO_2_ and H_2_O for ATP production, and the keto acids of the cycle produce the corresponding amino acids for the synthesis of bacterial cell materials (for more information on hydrocarbon utilization mechanisms refer to (25–28).

Careful analysis of the microbial dynamics (Figs. 1 and 2) consolidates the role of the bacterioflora in oil-consumption and highlights which taxa (taxon) in which heap at which phase might have played this role. To recall, during the adaptation/stabilization month of March, only limited proportions of oil have been lost (16–18%). The predominant taxa during this month in the undiluted and diluted heaps were affiliated within the genera: *Arthrobacter*, *Pseudomonas*, *Blastococcus*, *Kocuria*, *Acinetobacter*, *Stenotrophomonas* and *Nocardia*. One month later, i.e. end of April, chronologically with the maximum oil-consumption, most of those taxa did not predominate in the heaps. Instead, taxa affiliated with the genera: *Alkanindiges*, *Nocardioides*, *Micrococcus*, *Microbacterium*, *Cellulosimicrobium*, *Arthrobacter* and *Dietzia* (especially *D*. *papillomatosis*) took over the predominance. Therefore, these latter taxa should have been the major contributors to the measured oil-bioremediation. The only taxon which continued to predominate through the subsequent months was *Dietzia papillomatosis*, which confers on it a leading role in this specific oil-bioremediation process. This coordinates well with the finding that most of the studied strains tolerated up to 20% oil and propagated at those concentrations (Fig. 3)

Some genera were detected only in the months with rather moderate temperature, others were limited to the hot months and still others tended to show up in months with moderate and hot temperatures simultaneously. This implies that the hydrocarbonoclastic bacterial consortia in all the heaps consumed oil through the whole year. Scanning Figs. 1 and 2 from tops downward reveals that several minor taxa in all heaps took over the predominance with progressive bioremediation. Most prominent representative of such taxa was *Dietzia papillomatosis* (strain 8). The fact that those taxa tolerated high oil-concentrations (Fig. 3) coordinates with this predominance pattern and consolidates that they were major contributors to oil-removal in all the heavily polluted soil heaps.

The finding that taxa prevailing at the beginning do not remain so sheds some doubt in the use of of bioaugmentation as an approach for oil-bioremediation. This result simply implies that the organisms are mostly in a dynamic state. Proper microorganisms may be inoculated, but fail to remove the pollutant^32^. As recommended long ago^33^, oil-bioremediation should depend on the own indigenous microflora.

## Conclusions

Heavily oil-polluted sites need not to be diluted to enhance the activities of their indigenous oil-degrading bacteria. The high oil concentration selectively enriches such sites with bacterial strains capable of tolerating and biodegrading oil hydrocarbons. This study showed that these microorganisms occurred in relatively high numbers, and showed a striking diversity in their identities. These organisms are also active under oil-saturation conditions. However, the polluted soils should of course be kept moistened and well aerated. Other managements e.g. via N- and P-fertilization would still enhance the bacterial oil-removal.

## Methods

### Soil samples

Pristine (oil-free) desert soil samples were collected from Mishref, 14 km south of Kuwait City in the first of March 2017. Pristine garden soil was collected from the Botanical Garden of the Faculty of Science, Kuwait University, Khaldiyah, Kuwait. Part of the desert soil was mixed with 17.3%, w/w, of light Kuwaiti crude oil (National Oil Company) and used through the study as the oil-saturated sample. This concentration was the maximum oil-holding capacity of the desert soil as determined in preliminary experiments. Three 100 g portions were mixed with 25 g portions of oil, excess oil was decanted and the oil-holding capacity of the desert soil was determined gravimetrically. The other part of the pristine desert soil, as well as the pristine garden soil were used for diluting the oil-saturated soil. The oil-saturated soil was first kept the whole month of March (beginning of the experiment) on a glass plate in a protected area of the Botanical Garden as one heap exposed to the prevailing outdoor conditions. This treatment was proposed to provide the indigenous microbial community with a period of adaption to the high oil-concentration under open conditions.

### Oil-bioremediation in microcosms

Aliquots of the oil-saturated soil (OSS) were diluted either with pristine desert or pristine garden soils. One kg portions of this OSS were mixed with 0.25, 0.50, or 0.75 kg of the pristine soil. Through this text, the three dilutions with decreasing oil-concentrations are designated for ease as I, II and III, respectively. The OSS- and the I-, II- and III-heaps with total weights of 1, 1.25, 1.5 and 1.75 kg, respectively were inserted on glass plates to avoid contact with soil microorganisms below. Each heap was thoroughly mixed after moistening it with 10% water (w/w). The heaps were kept under open conditions in the Botanical Garden. They were moistened with equal amounts of water when necessary. At the ends of the adaptation month (March) and each of the subsequent 6 months, triplicate soil samples were collected for measurement of oil-consumption and for microbiological analysis as described below.

### Measurement of oil-consumption

As a rule, the soil heaps were thoroughly mixed weekly and immediately before each sampling using steel spatula. This guaranteed that the samples taken were reproducible and that the soil samples were well aerated. Oxygen is long known to be an essential requirement for the initial attack of the organism on the hydrocarbon substrate^34–37^. *In situ*, this treatment could be simulated by adopting conventional land-farming approaches. Three random samples were harvested from each heap; 1, 1.25, 1.5 and 1.75 g from the OSS-, I-, II- and III-heaps, respectively. This guaranteed that the extracted samples had in the beginning the same oil-concentration of 17.3%. The residual oil in each sample was recovered using three successive portions of 30 ml pentane. The combined extract was raised to 90 ml using pure pentane, and 1 µl was analyzed by Gas-Liquid Chromatography using an Aglient 7890 A GLC (USA) system equipped with FID, a DB-5 capillary column (Aglient Technologies, USA) and He as a carrier gas. The oven temperature started at 50 °C for 3 min, then rising at 3 °C/min to 80 °C, then rising at 8 °C/min to 256 °C, then rising at 30 °C/min to 330 °C, and holding at this temperature for 11 min. The amounts and percentages of the oil consumed were calculated in terms of the reduction values of total peak areas based on the total peak areas of the oil recovered from the abiotic control (similarly prepared but using autoclaved inocula).

As a preliminary test for the effectiveness of the bioremediation processes, oil-saturated soil samples at time zero and samples that had been subjected to bioremediation were filled into pots. Each pot was sown with 50 seeds of broad beans (*Vicia faba*). The soils were moistened with equal amounts of water and kept in the Green House. The percent values of germinating seeds were calculated. The seedlings in the pots were thinned out and left to grow for 6 weeks.

### Microbiological analysis

The soil heaps were well mixed before 3 random 1 g samples were collected from each heap for the analysis of the constituent hydrocarbonoclastic bacterial communities. The standard plating method using a solid mineral medium^38^ and crude oil vapor as a sole source of carbon and energy was used to count and isolate these microorganisms. The mineral medium consisted of (gl^−1^): 5.0 NaNO_3_, 0.56 KH_2_PO_4_, 0.86 Na_2_HPO_4_, 0.17 K_2_SO_4_, 0.37 MgSO_4_.7H_2_O, 0.007 CaCl_2_.H_2_O, 0.004 Fe (III) EDTA, 20.0 bacteriological agar; and trace element solution (25 ml l^−1^) consisting of (g l^−1^): 2.32 CuSO_4_.5H_2_O, 0.39 Na_2_MoO_4_.2H_2_O, 0.66 KI, 1.0 EDTA, 0.4 FeSO_4_.7H2O, 0.004 NiCl_2_.6H_2_O. The medium pH was adjusted to 7.

The composition of the different microbial communities was analyzed and their dynamics during bioremediation was monitored. Representatives of morphologically and microscopically identical colonies (colors, diameters, margins, consistencies, cell-shapes, motility, staining reactions, etc.) were subcultured and purified by streaking them on the above medium.

Individual pure isolates were characterized by comparing the sequences of their 16 S rRNA-coding genes with those of type strains in the GenBank database. Total genomic DNA of each purified strain was extracted with PrepMan Ultra Sample Preparation Reagent (Applied Biosystems, USA) and the 16 S rRNA-gene therein was amplified by polymerase chain reaction (PCR) using a Veriti Thermal Cycler (Applied Biosystems, USA) following a touch-down protocol of initial denaturation at 95 °C for 5 min, annealing temperature starting at 65 °C and decreasing by 1 °C every cycle to 55 °C, at which additional 12 cycles were carried out, denaturation was at 94 °C for 1 min, and primer extension at 72 °C for 1 min. The final extension was at 72 °C for 7 min. The PCR mixture consisted of puReTaq Ready-To-Go PCR Beads (Amersham Biosciences, UK), 1 µl (25 ng) of DNA template, 1 µl each of the universal primers GM5F (5′-CCT ACG GGA GGC AGC AG-3′) and 907 R (5′-CCG TCA ATT CMT TTG AGT TT-3′)^39^. The reaction volume was completed to 25 µl with molecular water (Sigma). The PCR products were purified using the QIA quick PCR purification kit (Qiagen, USA). Partial sequencing of the 16 S rRNA-genes was done using the BigDye version 3.1 Terminator Kit (Applied Biosystems, USA); 20 ng of the DNA template was added to 2 µl of the BigDye Terminator ready reaction mix; 2 µl of the 5X sequencing buffer, l µl primer either 907 R or GM5F, was added to the mixture, and the final volume was brought up to 10 µl with molecular water. Labeling was completed in the Veriti Thermal Cycler (Applied Biosystems, USA) using one cycle of 96 °C for l min, then 25 cycles of l min at 96 °C, 5 s at 50 °C and 4 min at 60 °C. DNA samples were processed in a 3130xl genetic analyzer (Applied Biosystems, USA). Sequencing analysis version 5.2 software (Applied Biosystems, USA) was used to analyze the results. Sequences were subjected to basic local alignment search tool analysis with the National Center for Biotechnology Information (NCBI; Bethesda, MD, USA) GenBank database^40^. The sequences were deposited in the GenBank under the accession numbers, MK161114-MK161220 (Supplementary Table S2).

### Oil-tolerance of pure isolates

Totally, 17 pure, hydrocarbonoclastic isolates were used in this experiment. Common inocula were prepared (1 loopful of bacterial biomass in 5 ml sterile water). Mineral medium^38^, 20 ml aliquots containing 1, 5, 10 or 20% crude oil as a sole carbon source were inoculated with 0.1 ml portions of the common inocula (≡10^6^ cells). The cultures were incubated on an electrical shaker, 180 rpm, at 30 °C for 5 days. Using the standard plating method with nutrient agar as a medium, the growth in terms of total CFU numbers was measured in each culture, as described above.

### Statistical analysis

Three determinations for each analysis were done and the mean values ± standard deviation values were calculated using Microsoft Excel 2007. The analysis of variance (ANOVA) and one-way analysis of covariance (ANCOVA) with time as the covariate, type of soil as the independent variable and number of bacteria or oil-consumption as the dependent variable were used to differentiate between the means of the tested parameters.

## Supplementary information

Supplementary information.
