# Supplementary material for: Bioremediation of soils saturated with spilled crude oil
Source: Sci Rep. 2020 Jan 24;10:1116. doi: 10.1038/s41598-019-57224-x (PMC6981149; doi:10.1038/s41598-019-57224-x)
Supplement: Supplementary file 1 — Supplementary information. [file 41598_2019_57224_MOESM1_ESM.pdf]

Bioremediation of soils saturated with spilled crude oil

Nedaa Ali[1], Narjes Dashti[1], Majida Khanafer[1], Husain Al-Awadhi[1] & Samir Radwan[2]

[1]Microbiology program, Department of Biological Sciences, Faculty of Science, Kuwait University, P.O.Box 5969, Safat 13060, Kuwait. [2]Permanent address: Von Einem Str. 25, 48159 Münster, Germany. Correspondence and requests for materials should be addressed to S. R. (email: samir.radwan@ku.edu.kw) and H.A. (email: husain.alawadhi@ku.edu.kw)

Table S1.

Minor hydrocarbonoclastic bacteria in oil-saturated soil (OSS) and OSS diluted (with pristine desert soil: I, 1 kg OSS + 0.25 kg; II, 1 kg OSS + 0.5 kg; III, 1 kg OSS + 0.75 kg) during bioremediation (shaded areas in Pie charts in Fig. 1 and 2).

End of month	Oil-saturated soil (OSS)	Diluted with pristine desert soil			Diluted with pristine garden soil		
		I	II	III	I	II	III
0 March	*Dietzia maris*	*Cellulomonas iranensis*	*Pseudomonas songnenensis*		*Pseudomonas songnenensis*	*Lentzea chajnantorensis*	
	Streptomyces gulbargensis	*Pseudomonas songnenensis*	*Streptomyces griseoflavus*			*Microbacterium paludicola*	
	Williamsia marianensis	*Rhodococcus gordoniae*	*Williamsia marianensis*			*Rhodococcus erythropolis*	
	Nocardioides albus	*Streptomyces griseoflavus*	*Actinotalea ferrariae*				
		Streptomyces scopiformis					
		Williamsia marianensis					
1 April	*Agromyces iriomotensis*	*Gordonia hankookensis*	*Streptomyces maoxianensis*	*Pseudomonas knackmussii*	*Arthrobacter agilis*	*Kocuria turfanensis*	*Arthrobacter echini*
	Bacillus circulans	*Pseudarthrobacter phenanthrenivorans*	*Streptomyces maoxianensis*	*Pseudomonas songnenensis*	*Arthrobacter ginsengisoli*		*Arthrobacter ginsengisoli*
	Gordonia hankookensis	*Streptomyces youssoufiensis*	*Streptomyces maoxianensis*	*Williamsia marianensis*			*Dietzia maris*
	Pseudomonas songnenensis	*Streptomyces indoligenes*					*Dietzia papillomatosis*
	Streptomyces alboflavus						*Kocuria turfanensis*

2	May			*Acinetobacter radioresistens*<br>*Kocuria dechangensis*<br>*Kocuria himachalensis*<br>*Planococcus rifietoensis*<br>*Pseudomonas songnenensis*<br>*Rhodococcus gordoniae*<br>*Williamsia marianensis*	*Dietzia maris*<br>*Cellulosimicrobium funkei*		*Nocardioides solisilvae*<br>*Streptomyces neyagawaensis*	
3	June	*Skermanella aerolata*<br>*Cellulomonas iranensis*		*Dietzia maris*	*Agrococcus baldri*<br>*Agrococcus citreus*<br>*Microbacterium paludicola*<br>*Nocardioides deserti*	*Agrococcus baldri*<br>*Agrococcus citreus*<br>*Cellulomonas bogoriensis*<br>*Cellulomonas hominis*<br>*Modestobacter versicolor*<br>*Skermanella rosea*<br>*Cellulomonas cellasea*		*Rhodococcus gordoniae*<br>*Cellulomonas iranensis*

4	July	*Aeromicrobium erythreum*	*Microbacterium oryzae*	*Kocuria flava*	*Dietzia cinnamea*		*Cellulomonas iranensis*	*Williamsia marianensis*
		Agrococcus citreus	*Microbacterium paludicola*	*Ornithinimicrobium humiphilum*	*Micrococcus aloeverae*		*Kocuria dechangensis*	*Nocardioides zeae*
		Cellulomonas flavigena	*Pseudomonas balearica*	*Noviherbaspirillum agri*	*Modestobacter versicolor*		*Kocuria himachalensis*	
		Exiguobacterium arabatum	*Pseudomonas luteola*		*Pseudomonas songnenensis*		*Krasilnikoviella muralis*	
		Kocuria flava			*Rhodococcus gordoniae*		*Nocardioides flavus*	
5	August	*Blastococcus saxobsidens*		*Xylanimicrobium pachnodae*	*Cellulomonas iranensis*	*Xylanimicrobium pachnodae*	*Bacillus koreensis*	*Blastococcus aggregatus*
		Pseudomonas balearica		*Rhodocista pekingensis*	*Microbacterium oryzae*		*Kocuria flava*	*Kocuria polaris*
					Nocardioides deserti		*Micrococcus endophyticus*	*Nocardioides deserti*
					Nocardioides solisilvae		*Skermanella stibiiresistens*	*Rhodocista pekingensis*
							Ornithinimicrobium humiphilum	
6	September	*Kocuria flava*			*Microbacterium aureliae*	*Agrococcus baldri*	*Cellulomonas iranensis*	
		Microbacterium lacusdiani			*Microbacterium paludicola*	*Blastococcus aggregatus*	*Geodermatophilus taihuensis*	
		Cellulomonas hominis			*Nocardioides flavus*	*Blastococcus colisei*	*Kocuria polaris*	
		Cellulomonas massiliensis				*Kocuria turfanensis*	*Nocardioides sediminis*	

		Microbacterium petrolearium
		Planococcus donghaensis
		Planomicrobium glaciei

Table S2.

Sequencing of 16S rRNA-gene of hydrocarbonoclastic bacteria isolated from oil-saturated desert soil (OSS) and OSS diluted with pristine desert and garden soils.

Isolate designation	Total bases	Nearest Gene Bank match (accession no.)	Similarity %	Bases compared	Accession No.
HSC-3	498	*Streptomyces gulbargensis* (FN598542)	100	498/498	MK161114
HSC-4	532	*Pseudomonas songnenensis* (NR_148295)	100	532/532	MK161115
HSC-5	496	*Pseudomonas balearica* (NR_025972)	99	502/505	MK161116
HSC-20	533	*Agromyces iriomotensis* (NR_133690)	99	541/545	MK161117
HSC-23	491	*Bacillus circulans* (NR_118445)	99	497/500	MK161118
HSC-28	520	*Georgenia daeguensis* (NR_117960)	99	524/526	MK161119
HSC-30	477	*Acinetobacter radioresistens* (NR_114074)	99	483/486	MK161120
HSC-35	502	*Citricoccus yambaruensis* (NR_132666)	99	512/517	MK161121
HSC-45	516	*Skermanella aerolata* (NR_043929)	99	526/531	MK161122
HSC-48	517	*Exiguobacterium arabatum* (FM203124)	99	529/535	MK161123
HSC-56	510	*Xylanimicrobium pachnodae* (NR_024956)	99	522/528	MK161124
HSC-61	504	*Aeromicrobium erythreum* (NR_024846)	99	510/513	MK161125
HSC-75	515	*Blastococcus saxobsidens* (NR_117019)	99	517/518	MK161126
HSC-81	526	*Georgenia soli* (NR_116959)	99	530/532	MK161127
HSC-87	507	*Cellulomonas massiliensis* (NR_125601)	99	513/516	MK161128
HSC-506	516	*Streptomyces alboflavus* (NR_044151)	100	516/516	MK161129
HSC-511	518	*Micromonospora soli* (NR_146360)	99	520/521	MK161130
HSC-515	515	*Cellulomonas hominis* (NR_029288)	100	515/515	MK161131
DSA-89	509	*Streptomyces scopiformis* (NR_114403)	99	519/524	MK161132
DSA-98	513	*Pseudarthrobacter phenanthrenivorans* (NR_074770)	99	519/522	MK161133
DSA-100	513	*Nocardioides solisilvae* (NR_136850)	99	515/516	MK161134
DSA-102	501	*Nocardioides deserti* (KY436429)	99	503/504	MK161135
DSA-104	509	*Gordonia hankookensis* (NR_104507)	99	513/515	MK161136
DSA-105	477	*Streptomyces youssoufiensis* (NR_116980)	98	495/505	MK161137
DSA-111	414	*Nocardioides aquiterrae* (NR_025261)	99	416/417	MK161138
DSA-114	509	*Arthrobacter subterraneus* (KY436432)	99	513/515	MK161139
DSA-118	504	*Aeromicrobium massiliense* (NR_125588)	99	510/513	MK161140
DSA-124	504	*Gordonia amicalis* (NR_028735)	99	516/522	MK161141
DSA-134	525	*Pseudomonas luteola* (NR_114215)	99	539/546	MK161142
DSA-146	517	*Kocuria flava* (NR_044308)	100	517/517	MK161143
DSA-155	499	*Cellulomonas iranensis* (NR_024914)	99	503/505	MK161144
DSA-162	495	*Micrococcus flavus* (NR_043881)	99	497/498	MK161145
DSA-516	507	*Streptomyces indoligenes* (NR_149274)	99	515/519	MK161146
DSA-517	514	*Pseudonocardia halophobica* (NR_116193)	99	520/523	MK161147
DSB-163	506	*Streptomyces alfalfae* (NR_147713)	99	510/512	MK161148
DSB-165	503	*Kocuria dechangensis* (NR_137239)	99	511/515	MK161149
DSB-166	501	*Blastococcus aggregatus* (NR_114864)	100	501/501	MK161150
DSB-169	501	*Arthrobacter tumbae* (NR_042078)	100	501/501	MK161151

DSB-170	494	*Streptomyces griseoflavus* (KU720585)	100	494/494	MK161152
DSB-173	495	*Arthrobacter echini* (NR_148833)	99	503/507	MK161153
DSB-181	484	*Williamsia marianensis* (NR_118613)	99	486/487	MK161154
DSB-182	496	*Kocuria himachalensis* (LC113906)	99	499/501	MK161155
DSB-183	474	*Planococcus rifietoensis* (MF417406)	98	491/500	MK161156
DSB-192	502	*Dietzia maris* (NR_118596)	100	502/502	MK161157
DSB-196	515	*Kocuria polaris* (KX959605)	100	515/515	MK161158
DSB-197	504	*Roseomonas oryzae* (NR_137403)	99	506/507	MK161159
DSB-202	512	*Dietzia papillomatosis* (NR_116687)	100	512/512	MK161160
DSB-209	444	*Azospirillum rugosum* (NR_042582)	99	450/453	MK161161
DSB-525	511	*Actinotalea ferrariae* (NR_118034)	99	517/520	MK161162
DSB-529	516	*Streptomyces maoxianensis* (NR_148303)	99	518/519	MK161163
DSB-533	489	*Noviherbaspirillum agri* (KX129821)	97	521/537	MK161164
DSC-229	520	*Arthrobacter phenanthrenivorans* (NR_042469)	99	524/526	MK161165
DSC-235	430	*Pseudomonas knackmussii* (NR_117756)	99	436/439	MK161166
DSC-236	495	*Microbacterium aerolatum* (NR_028944)	99	499/501	MK161167
DSC-238	525	*Georgenia satyanarayanai* (NR_117051)	99	527/528	MK161168
DSC-243	493	*Dietzia cinnamea* (NR_116686)	100	493/493	MK161169
DSC-244	521	*Cellulosimicrobium funkei* (NR_042937)	99	527/530	MK161170
DSC-249	525	*Arthrobacter ginsengisoli* (KF212463)	99	527/528	MK161171
DSC-254	530	*Agrococcus citreus* (NR_041542)	99	534/536	MK161172
DSC-261	525	*Micrococcus endophyticus* (KY933306)	99	527/528	MK161173
DSC-267	529	*Micrococcus aloeverae* (NR_134088)	99	531/532	MK161174
DSC-268	518	*Modestobacter versicolor* (NR_042368)	100	518/518	MK161175
DSC-279	523	*Krasilnikoviella muralis* (LC148843)	99	531/535	MK161176
DSC-284	524	*Microbacterium oryzae* (NR_117527)	99	530/533	MK161177
DSC-290	520	*Microbacterium aureliae* (NR_152652)	99	524/526	MK161178
DSC-543	513	*Alkanindiges hongkongensis* (NR_115179))	99	525/531	MK161179
DSC-544	509	*Cellulomonas cellasea* (NR_037077)	99	511/512	MK161180
DSC-556	496	*Nocardioides flavus* (AF005014)	98	514/523	MK161181
GSA-299	511	*Micrococcus cohnii* (NR_117194)	99	519/523	MK161182
GSA-300	521	*Streptomyces yangpuensis* (NR_148776)	99	523/524	MK161183
GSA-304	493	*Arthrobacter agilis* (NR_026198)	99	515/526	MK161184
GSA-309	427	*Cellulomonas flavigena* (NR_074490)	98	443/451	MK161185
GSA-316	523	*Microbacterium lacusdiani* (KP986565)	99	529/523	MK161186
GSA-328	526	*Agrococcus baldri* (NR_041543)	99	532/535	MK161187
GSA-329	400	*Cellulomonas bogoriensis* (NR_114941)	99	412/418	MK161188
GSA-337	507	*Skermanella rosea* (NR_152076)	100	507/507	MK161189
GSA-356	508	*Rothia endophytica* (NR_109752)	99	516/520	MK161190
GSA-362	516	*Planomicrobium glaciei* (NR_044384)	99	520/522	MK161191
GSA-366	507	*Microbacterium petrolearium* (NR_134087)	99	513/516	MK161192
GSA-368	509	*Georgenia sediminis* (NR_109690)	98	529/539	MK161193
GSA-369	411	*Xylanimonas cellulosilytica* (NR_074544)	99	421/426	MK161194
GSA-370	520	*Planococcus donghaensis* (NR_044073)	99	524/526	MK161195
GSA-372	443	*Blastococcus colisei* (NR_149176)	99	455/461	MK161196

GSB-381	508	*Stenotrophomonas rhizophila* (NR_121739)	97	536/550	MK161197
GSB-382	525	*Lentzea chajnantorensis* (NR_149214)	99	527/528	MK161198
GSB-383	514	*Rhodococcus erythropolis* (KJ476725)	99	520/523	MK161199
GSB-395	523	*Streptomyces neyagawaensis* (NR_025868)	66	525/526	MK161200
GSB-409	530	*Kocuria turfanensis* (NR_043899)	99	534/536	MK161201
GSB-422	450	*Skermanella stibiiresistens* (NR_117972)	96	486/504	MK161202
GSB-424	542	*Bacillus persicus* (KY436466)	99	546/548	MK161203
GSB-425	523	*Microbacterium paludicola* (NR_114939)	99	525/526	MK161204
GSB-431	499	*Geodermatophilus taihuensis* (NR_109679)	98	523/535	MK161205
GSB-433	514	*Nocardioides sediminis* (NR_044228)	99	530/538	MK161206
GSB-588	510	*Cellulosimicrobium marinum* (NR_146662)	99	512/513	MK161207
GSB-590	511	*Pseudonocardia kongjuensis* (NR_025384)	100	511/511	MK161208
GSC-439	515	*Nocardia fluminea* (NR_117325)	100	515/515	MK161209
GSC-441	522	*Arthrobacter bambusae* (NR_133968)	99	426/528	MK161210
GSC-443	525	*Streptomyces fulvissimus* (KY820858)	99	527/528	MK161211
GSC-444	501	*Aeromicrobium ginsengisoli* (NR_041384)	98	517/525	MK161212
GSC-464	392	*Arthrobacter aurescens* (KJ476726)	99	394/395	MK161213
GSC-468	508	*Rhodococcus gordoniae* (AB915625)	99	518/523	MK161214
GSC-469	458	*Microbacterium ginsengiterrae* (NR_116483)	99	460/461	MK161215
GSC-490	480	*Rhodocista pekingensis* strain 3-p (NR_028855)	98	500/510	MK161216
GSC-492	506	*Nocardioides albus* (KY436468)	99	514/518	MK161217
GSC-500	547	*Moraxella osloensis* (KT989843)	100	547/547	MK161218
GSC-603	515	*Ornithinimicrobium humiphilum* (KT989854)	99	525/530	MK161219
GSC-606	489	*Nocardioides zeae* (NR_134102)	98	509/519	MK161220

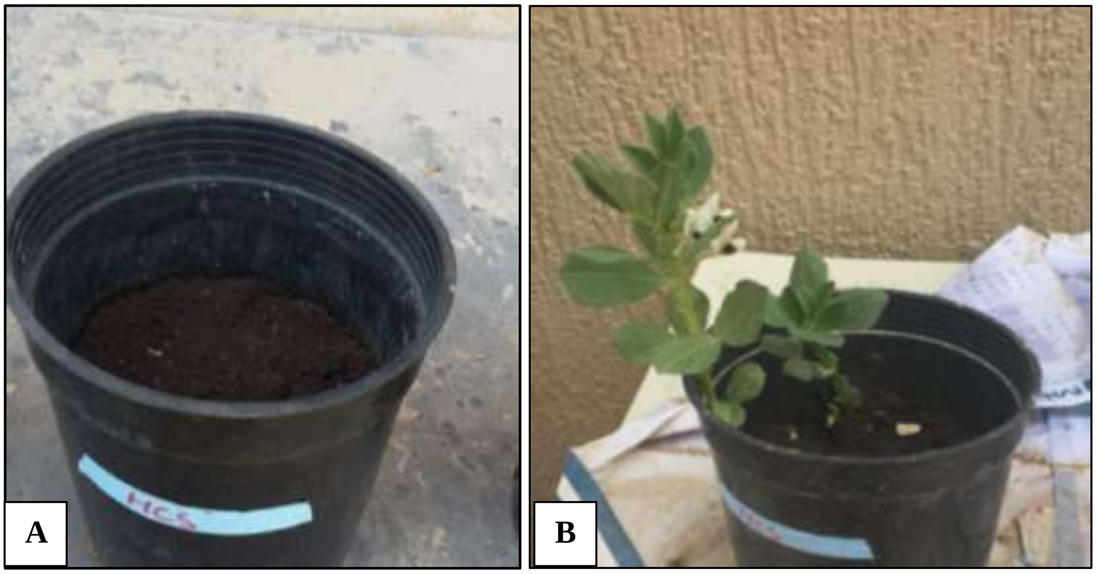

Fig. S1 Suitability of oil-saturated soil for cropping of broad beans..

A, Unbioremediated; B, 6-months bioremediated soil.
